# The impact of restrictions on psychological outcomes in patients with inflammatory bowel disease on biological treatment during the coronavirus pandemic in Norway

**DOI:** 10.1007/s11136-022-03254-4

**Published:** 2022-09-20

**Authors:** Randi Opheim, Kristian Marling Moum, Milada Cvancarova Småstuen, Bjørn Moum

**Affiliations:** 1 Department of Gastroenterology, Oslo University Hospital, Nydalen, Postboks 4959, 0424 Oslo, Norway; 2 Institute of Health and Society, University of Oslo, Blindern, Postbox 1130, 0318 Oslo, Norway; 3 Faculty of Health Science, University of Southern Denmark, Odense, Denmark; 4 Faculty of Health Science, Oslo Metropolitan University, Oslo, Norway; 5 Department of Gastroenterology, Oslo University Hospital, Oslo, Norway; 6 Institute of Clinical Medicine, University of Oslo, Oslo, Norway

**Keywords:** Anxiety, COVID-19, Depression, Inflammatory bowel disease, Patient-reported outcome, Quality of health care

## Abstract

**Purpose:**

The coronavirus (COVID-19) pandemic restrictions have led to changes in the follow-up routine of patients in outpatient clinics at hospitals in Norway. The purpose of this study was to assess possible associations between psychological health and concerns regarding COVID-19 societal and hospital restrictions in patients with inflammatory bowel disease on biological therapy.

**Methods:**

Patients with IBD (≥ 18 years) undergoing biological treatment (TNF-alpha inhibitor, ustekinumab, vedolizumab) for IBD were recruited from an IBD outpatient clinic in Norway. Data were collected through self-report, including questions covering concerns regarding their disease, medical therapy, and follow-up during the pandemic, Patient Health Questionnaire–9 (PHQ-9) and Generalized Anxiety Disorder–7 questionnaire (GAD-7). Multiple logistic regression with backward conditional selection was fitted to examine associations between patients’ depression and anxiety levels and their concerns about COVID-19 restrictions, controlled for sociodemographic and disease-related factors.

**Results:**

Five-hundred and six patients were included in this study. General condition, self-isolation, employment status, fear of visiting the hospital, and changes to patients’ appointments made by the hospital were independently associated with higher levels of depression. Female gender, experiencing symptoms of COVID-19, self-isolation, experiencing an increased risk of COVID-19 because of IBD, being afraid to visit the hospital because of COVID-19 restrictions, and having their appointment cancelled due to COVID-19 were independently associated with higher anxiety levels.

**Conclusion:**

Concerns about physical health and societal and hospital restrictions were associated with anxiety and depression in patients with IBD undergoing biological treatment. The findings will help facilitate healthcare services for patients with IBD in outpatient clinics and develop guidelines for follow-up.

## Plain English summary

*Why is the study needed*: COVID-19 pandemic has led to substantial changes in routine clinical follow-up because of societal and hospital restrictions. Patients with inflammatory bowel disease (IBD) undergoing biological therapy may be particularly vulnerable because of the increased risk of infection and compromised immune response to the virus. IBD patients’ psychological health and concerns regarding COVID-19 societal and hospital restrictions have not been studied well.

*What is the key problem/issue/question this manuscript addresses?* To facilitate healthcare services for patients with IBD during the pandemic, knowledge of how restrictions may impact psychological health is required.

*What is the main point of the study?* Easy access to mail and telephone support and sufficient information are required to improve IBD patients’ psychological well-being and quality of care.

*What is the main results and what do they mean*: Concerns of patients with IBD about their physical health and societal and hospital restrictions were associated with impaired psychological health. Preventive factors included structural elements such as easy access to mail and telephone support and sufficient information and disease advice from physicians regarding IBD.

## Introduction

Patients with inflammatory bowel disease (IBD), which includes Crohn’s disease (CD) and ulcerative colitis (UC), have experienced substantial changes in the routine management of their clinical follow-up during the coronavirus disease 2019 (COVID-19) [1, 2]. IBD that frequently requires treatment with immunomodulatory medicines and biological therapy can increase the risk of infection and compromise the immune response to the virus. At the beginning of the pandemic in 2020, and before the vaccines had been introduced, governmental and hospital restrictions were initiated in order to prevent the spread of infection. These restrictions included social distancing, forced/involuntary home isolation, closure of schools, universities and other public facilities. There was however little or no information or spesific recommandations for precautions for vulnerable patient groups. During the pandemic, the need for information and close follow-up has been particularly important for patients [1]. Alteration in the patients’ scheduled follow-up due to restrictions may lead to concern regarding their disease activity and the risk of becoming sick. In addition to providing medical care and disease management, there is a need to be attentive to patients’ worries and concerns in response to pandemic restrictions and to monitor if these restrictions cause variations in disease expression and psychological health, warranting closer follow-up.

Patients with IBD tend to have significantly poorer health-related quality of life compared to healthy individuals [3]. To identify and respond to the psychological needs of patients in clinical care may be of importance for their health-related quality in life [4]. Quality of care has been described by the World Health Organization as “the extent to which health care services provided to individuals and patient populations improve desired health outcomes” [5]. Quality must be continually measured and monitored to drive improvement, which relies on accurate, timely, and actionable data. The preventive aspects of care, such as the management of patients’ psychological well-being are being increasingly identified as a necessary component of care [6].

Studies have reported that symptoms of anxiety and depression and self-reported stress are common psychological reactions to the COVID-19 pandemic in a healthy population [7–9]. A Norwegian study revealed a twofold to threefold increase in depressive and anxiety symptoms in the general adult population early in the pandemic compared with pre-pandemic samples, and participants who socially distanced themselves reported substantially higher symptoms than their counterparts. Social distancing is likely to increase loneliness [7, 10]. Several studies have examined anxiety and depression in populations with IBD before the pandemic [11, 12]. Uncertainty, lack of information, concerns, and worries about medical treatment were associated with higher rates of anxiety and depression [13]. A recent finding related to the biological treatment of IBD patients during the COVID-19 pandemic reported that these patients were more cautious than others, such as being voluntarily isolated and on sick leave [14].

This study aimed to assess possible associations between IBD patients’ psychological health and experience and concerns regarding COVID-19 societal and hospital restrictions, sociodemographic factors, and disease-related factors.

## Methods

### Study design and population

Participants were consecutively included when they met up for their scheduled treatment and/or follow-up at a tertiary IBD outpatient clinic in Oslo, Norway, during the time period June 18th to September 18th, 2020. The inclusion criteria were adult patients with IBD (≥ 18 years) who received biological treatment (TNF-alpha inhibitor, ustekinumab, vedolizumab) for Crohn’s disease or ulcerative colitis. Additionally, participants had to be able to read and understand Norwegian and provide written informed consent.

#### Data collection

Sociodemographic variables were self-reported by patients, and they included age, gender, marital status, educational level, and employment status, including being on sick leave from work or temporarily laid off from work or school. Disease-related data were collected by interview and included the type of diagnosis, current IBD medication, any history of surgery for IBD, years since diagnosis. Furthermore, the patients were asked to assess their general condition during the last 24 h (level of activity, energy level, and overall well-being) and the responses were categorised as follow: unaffected, slightly reduced, poor, and very poor. The participants were asked if they had experienced COVID-19 symptoms, including whether they had been tested.

Furthermore, the participants were asked about their treatment adherence (i.e., if they voluntarily stopped IBD medication), adherence to societal and hospital restrictions (yes/no), concerns regarding these restrictions (yes/no), whether they were self-isolated and stayed away from their family and friends (yes/no), if they were concerned regarding their disease, and medical treatment for IBD related to COVID-19 (yes/no). Additionally, questions were asked about how they experienced the information provided by a physician and other healthcare staff during the pandemic, as well as what was their preferred type of follow-up (i.e., physical, telephone, e-mail, video consultation).

#### Patient health questionnaire–9 (PHQ-9)

The PHQ-9 assesses symptoms of depression [15]. The questionnaire consists of nine items, each of which is scored on a 4-point Likert scale, with total scores ranging from 0 to 27. Higher scores indicate greater depression severity, and a score of 10 or above indicates major depression [16]. The PHQ-9 has been validated in an IBD population and has demonstrated good psychometric properties [17]. The internal consistency of this scale in our study population was good, with a Cronbach’s α of 0.87.

#### Generalized anxiety disorder–7 (GAD-7)

The GAD-7 was used to measure anxiety.[18] The questionnaire consists of seven items and is scored on a four-point Likert-type scale, with scores ranging from 0 to 21. Higher scores indicate greater anxiety severity, and a score ≥ 8 indicates that anxiety is clinically important [19]. The questionnaire has been validated in Norwegian and IBD populations and has shown good psychometric properties, [17–19] with a Cronbach’s α of 0.89 in our study population.

### Statistical analyses

Descriptive variables were described as mean and standard deviation (SD) for continuous variables. Categorical variables were presented as counts and percentages. The prevalence of depression and anxiety is presented as the estimated proportion with 95% confidence interval (CI).

Logistic regression models were fitted to assess possible associations between depression and anxiety (defined as yes = cut-off ≥ 10 points on the PHQ-9 and  ≥ 8 points on the GAD-7) and selected possible predictors factors. The evaluated variables were sociodemographic variables (age in categories < or > 50 years), gender (in categories male vs. female), civil status (dichotomized into married or divorced/unmarried/widower), work status (working or student, sick leave /temporarily laid off or disability benefit/pensioner), education level (elementary school, upper secondary or university level); disease-related variables (type of diagnosis, general condition (dichotomized into unaffected (unaffected, slighly reduced) or affected (poor, very poor)), number of years since diagnosis); and questions regarding COVID-19 restrictions and healthcare needs. Questions about COVID-19 were included in the analyses when at least 20 individuals selected the smallest available response category to ensure there was enough heterogeneity in the data to estimate with sufficient precision and to ensure numerical stability of the estimates.

Multiple logistic regression with backward conditional variable selection was used. All described possible predictive factors were included in the initial model and variables with the lowest *p*-values in the previous step were included in the next step until only variables with *p*-values < 0.01 were retained in the final model. The strength of the association between the outcome and the evaluated possible predictive factors was expressed as an odds ratios (OR) with 95% confidence intervals (CI). To avoid overfitting, the final models were restricted to at most nine covariates for the PHQ-9 and six covariates for GAD-7. As the number of remaining statistically significant covariates in the final step of regression modeling was smaller than 9 and 6, respectively, no further model selection was performed. [20, 21] All analyses were considered exploratory, so no correction for multiple testing was performed, and *p*-values < 0.05 were considered statistically significant. All analyses were performed using IBM SPSS Statistics for Windows version 26.0 (IBM Corp., Armonk, NY, USA, released in 2019).

## Results

### Study sample

A total of 522 patients with IBD were included in the study after providing written informed consent. Of these, 16 patients did not use biological treatments or did not complete the questionnaires, and were therefore excluded. All the remaining 506 questionnaires could be evaluated (response rate: 97%). Of the 506 analyzed patients, 217 (42.9%) were women, with a mean age of 40.8 years (SD 14.7); 199 (39. 3%) had UC, and 307 (60.7%) had CD. Detailed characteristics of the study sample are presented in Table 1.

**Table 1:** Characteristics of inflammatory bowel disease patients on biological treatment (*N* = 506)

	IBD (N = 506)	UC (n = 199)	CD (n = 307)
Socio-demographic characteristics			
Age			
< 50	374 (74.1)	155 (78.3)	219 (71.3)
≥ 50	131 (25.9)	43 (21.7)	88 (28.7)
Missing		1	
Gender			
Male	289 (57.1)	124 (62.3)	165 (53.7
Female	217 (42.9)	75 (37.7)	142 (46.3)
Education (*n*, %)			
Elementary School	22 (4.4)	6 (3.0)	16 (5.2)
Upper Secondary	146 (28.9	57 (28.8)	89 (29.0)
University	337 (66.7)	135 (68.2)	202 (65.8)
Missing		1	
Civil Status (*n*, %)			
Married	316 (62.6)	142 (71.7)	174 (56.7)
Divorced/unmarried/widowed	189 (37.4)	56 (28.3)	133 (43.3)
Missing		1	
Working Status (*n*, %)			
Working or Student	379 (75.0)	154 (77.8)	225 (73.3)
Sick Leave/Temporarily Laid Off	38 (7.5)	18 (9.1)	20 (6.5)
Disability Benefit/Pensioner	88 (17.4)	26 (13.1)	62 (20.2)
Missing		1	
Clinical characteristics			
Years Since Diagnosis			
Median (range)	11 (1–55)	9 (1–49)	13 (1–55)
Missing			4
Gastrointestinal surgery (*n*, %)			
Never		181 (91.0)	143 (46.6)
Once		5 (2.5)	49 (16.0)
Twice or more		13 (6.5)	115 (37.5)
Biological treatment in combination with AZA or steroids	63 (12.1)	30 (15.1)	33 (10.7)
General condition (*n*, %)			
Unaffected	303 (59.9)	131 (65.8)	172 (56.0)
Reduced	203 (40.1)	68 (34.2)	135 (44.0)
Depression (PHQ-9)			
Mean (SD)	5.55 (5.04)	5.35 (4.92)	5.69 (5.11)
Range	0–24	0–21	0–18
Missing	8	3	5
Anxiety (GAD-7)			
Mean (SD)	3.29 (4.00)	3.52 (4.06)	3.14 (3.96)
Range	0–21	0–18	0–21
Missing	9	1	8

*IBD* inflammatory bowel disease; *UC* ulcerative colitis; *CD* Crohn's disease; *AZA* Azathioprine; *PHQ*-9 Patient Health Questionnaire 9; *GAD* -7 General Anxiety Disorder-7

Continuous variables were estimated by student’s *t*-test and the Mann Whitney *U* test

Table 2 describes the IBD patients’ experience of restrictions due to the pandemic. Eighty patients (15.8%) thought they had an increased risk of being infected with COVID-19 because of IBD, and one-third voluntarily self-isolated. Thirty-eight patients (7.6%) were afraid of going to the hospital and 20 patients (4.0%) cancelled their appointment. The hospital re-scheduled from physical appointment to a telephone follow-up for 75 patients (15%). Bivariate analyses revealed that a significantly higher proportion of women compared to men reported symptoms of COVID-19, 48 (22.2%) vs. 39 (13.5%), respectively. In addition, a significanlty higher proportion of patients < 50 years reported symptoms of COVID-19, compared to those >  = 50 years, *n* = 73 (19.6%) vs. *n* = 14 (10.7%), respectivily. Furthermore, a significantly higher proportion of women (38.5%) compared to men (27.7%) reported voluntarily self-isolation. There were no statistically significant differences between patients with ulcerative colitis and Crohn’s disease.

**Table 2:** IBD patient reported worries and concerns about the COVID-19 restrictions

	IBD (*n* = 506)
Voluntarily Stopped Treatment (*n*, %)	
Yes	7 (1.4)
Missing	3
Symptoms of COVID-19 (*n*, %)	
Yes	87 (17.2)
Missing	
Voluntarily self-isolated (*n*, %)	
Yes	161 (32.3)
Missing	8
Thought they had an increased risk of being infected of COVID-19 because of IBD (*n*, %)	80 (15.8%)
Missing	1
Was afraid of going to the hospital because of COVID-19 restrictions (*n*, %)	38 (7.6%)
Missing	3
Cancelled their appointment (*n*, %)	20 (4.0%)
Missing	3
Had their physical appointment re-scheduled to a telephone follow-up (*n*, %)	75 (15)
Missing	7

*IBD* inflammatory bowel disease

### PHQ-9 and GAD-7 scores

Of the IBD patients, 90 patients (18.1%) met the criteria for major depression (PHQ-9 score ≥ 10), and 62 patients (12.5%) met the criteria for anxiety (GAD-7 score ≥ 8). Of those with a score ≥ 10 on PHQ-9, 51.1% were women; and of those with a score ≥ 8 on GAD-7, 61.3% were women. The mean PHQ-9 score was 5.52, 95% CI [5.07, 5.96]. Women had a significantly higher PHQ-9 scores compared to men (6.19, 95% CI [5.51, 6.88] vs. 5.05, 95% CI [4.42, 5.59], respectively). The mean GAD-7 score was 3.30, 95% CI [2.94, 3.65] for all 506 patients (Table 2). Women had a significantly higher GAD-7 score compared to men (4.13, 95% CI [3.53, 4.72] vs. 2.67, 95% CI [2.25, 3.08], respectively). Forty eight patients met the criterion for both anxiety and depression based on the cut-off scores.

### Factors associated with depression

Logistic regression analyses were used to assess the strength of associations between major depression and sociodemographic and disease-related factors, questions regarding COVID-19 restrictions, and healthcare needs (Table 3). When adjusted for possible predictive factors, general condition, self-isolation, employment status, being afraid to go to the hospital, and whether the hospital made changes to patients’ appointments were all independently and significantly associated with higher depression levels. Compared to patients with unaffected general condition, patients with reduced condition were over five times more likely to be depressed (OR = 5.24, 95% CI [3.00, 9.19]). Those who chose to self-isolate were 2.7 times more likely to be depressed compared to those who did not (OR = 2.70, 95% CI [1.56, 4.70]).

**Table 3:** Variables associated with major depression (PHQ-9 ≥ 10). Multiple logistic regression

	OR	95% CI	*p*-value
General condition			
Unaffected (ref)	1		
Reduced	5.24	3.00–9.19	< 0.001
Self-isolation (voluntarily)			
No (ref)	1		
Yes	2.70	1.56–4.70	< 0.001
Work status			
Employed/student (ref)	1		
Sick leave/temporarily laid off	3.80	1.69–8.51	0.001
Disability benefit/pensioner	0.89	0.43–1.85	0.759
Afraid to go to the hospital because of restrictions due to COVID-19?			
No (ref)	1		
Yes	2.62	1.08–6.38	0.033
Did the hospital change your consultation at the hospital to a phone consultation?			
No (ref)	1		
Yes	0.29	0.12–0.72	0.007

*OR* odds ratio, *Ref* reference category

Patients on sick leave or temporarily laid off were almost four times more likely to be depressed compared to patients who were employed or students (OR = 3.80, 95% CI [1.69, 8.51]). However, there were no differences in the odds of developing clinical depression between employed patients and those with disability benefits or pensioners.

Being afraid of going to the hospital had a significant impact on patients’ odds of clinical depression. Those who were afraid of going to the hospital were 2.6 times more likely to be depressed compared to those who were not afraid (OR = 2.62, 95% CI [1.08, 6.38]).

Changing the type of consultation from physical consultation to phone consultation greatly diminished patients’ odds of being depressed. The patients who had their consultation changed to phone consultation had about 70% reduced odds of depression compared to those who did not experience such changes (OR = 0.29, 95% CI [0.12, 0.72]).

### Factors associated with anxiety

Logistic regression analyses were used to assess the association between anxiety and sociodemographic and disease-related factors, questions regarding COVID-19 restrictions, and healthcare needs (Table 4). The multiple logistic regression model revealed that female gender, having symptoms of COVID-19, self-isolation, perceived themselves as being more likely to get infected with COVID-19 because of IBD, as well as being afraid to go to the hospital because of COVID-19 restrictions, and having their appointment cancelled due to fear of getting infected were all independently and significantly associated with higher levels of anxiety.

**Table 4:** Variables associated with anxiety (GAD-7 ≥ 8). Multiple logistic regression

	OR	95% CI	*p*-value
Gender			
Male (ref)	1		
Female	2.01	1.08–3.74	0.027
Symptoms of COVID-19			
No (ref)	1		
Yes	2.79	1.41–5.53	0.003
Self-isolation (voluntarily)			
No (ref)	1		
Yes	3.60	1.93–6.74	< 0.001
Increased risk of being infected with COVID-19 because of IBD			
No (ref)			
Yes	1		
Don’t know	2.33	1.00–5.44	0.050
	2.78	1.33–5.81	0.006
Afraid to go to the hospital because of restrictions due to COVID-19?			
No (ref)	1		
Yes	3.29	1.38–7.82	0.007
Have you cancelled your consultation due to a fear of getting infected with COVID-19?			
No (ref)	1		
Yes	2.89	0.86–9.69	0.085

*OR* odds ratio, *Ref* reference category

Women were twice as likely to reach high levels of anxiety compared to men (OR = 2.01, 95% CI [1.08, 3.74]). Patients who experienced COVID-19 symptoms were almost three times more likely to be anxious compared to those who did not experience it (OR = 2.79, 95% CI [1.41, 5.53]).

Patients who perceived themselves as being more likely to get infected with COVID-19 had more than two times higher odds of being anxious (OR = 2.33[1.0, 5.44] and OR = 2.78[1.33, 5.81], for those who perceived themselves at higher risk and those who did not know if they were at such a risk, respectively. Being afraid of going to the hospital increased the odds of being anxious by more than threefold compared to patients who were not afraid (OR = 3.29, 95% CI [1.38, 7.82]).

Patients who cancelled their IBD consultation due to fear of becoming infected were more than twice as likely to be anxious compared to patients without such concern (OR = 2.89, 95% CI [0.86, 9.69]; however, the odds did not reach the level of statistical significance (*p* = 0.085).

### Discussion

Our results showed that, overall, a low proportion of IBD patients were worried about their treatment and follow-up at the hospital, and a high proportion felt safe at the hospital. However, we found several COVID-19-related factors independently associated with anxiety and depression.

One-fifth of patients reported major symptoms of depression. Our finding is in accordance with a rate of 22.5% in a newly published study that assessed depression with PHQ-9 in a hospital-based sample of IBD patients in the United Kingdom during the pandemic [22]. In studies assessing depression with PHQ-9 in IBD populations before the pandemic, a prevalence between 25% and 34% was found [23, 24]. Only 12.5% reported anxiety in our study, which is somewhat lower than findings in a hospital-based cohort in the United Kingdom that found a prevalence of 18% [22]. A study assessing anxiety with GAD-7 in an IBD population in 2018 found a prevalence of 21% [24]. The mean levels of depression and anxiety in our study were however lower than the estimates from a study conducted among the general Norwegian population simultaneously during the pandemic [15].

Systematic reviews and meta-analyses have shown that anxiety and depression are prevalent among patients with IBD in the pre-pandemic samples [11, 12]. Comorbid anxiety and depression may affect the IBD patients’ health-related quality of life and their ability to manage living with IBD. A systematic literature review revealed that a systematic approach to screening for anxiety and depression is not common in IBD quality of care, leaving the patients non-medical needs untreated [4]. To facilitate healthcare services for patients with IBD during the pandemic, knowledge of how restrictions may impact psychological health is needed to meet the patients’ non—medical needs and to include mental health professionals when needed.

An impaired general condition was the strongest predictor of depression. Patients in our study who had reported a decreased general condition were over five times more likely to be depressed than patients with unaffected general condition. General conditions and experiencing COVID-19 symptoms were the only disease-related factors associated with adverse psychological health outcomes. Both the general condition and symptoms of COVID-19 were self-assessed by the patients, and thus their subjective assessments of feeling sick. We assume that both physical and psychological factors are considered when assessing one’s level of activity, energy level, and overall well-being. These factors are also considered symptoms of depression, which may help explain the strong statistical association between these variables and the outcome.

Self-isolation, fear of visiting the hospital, and fear of contracting COVID-19 were all factors strongly associated with anxiety and depression. The same factors were found in a Portuguese study that used an online survey to assess the effects of the pandemic on disease and psychological outcomes among patients with IBD [25]. Systematic reviews have revealed that anxiety and depression rates in patients with IBD were associated with impaired health-related quality of life and higher healthcare use [3, 26]. This may indicate that IBD patients have unmet needs related to support for psychological issues during follow-up from healthcare providers. We found that changing from a physical hospital appointment to telephone consultation was a predictive factor for depression in our patients with IBD. The patients who had their consultation changed to telephone consultation had about 70% reduced odds of depression compared to those who did not experience such changes. To feel safe for patients, it is important to provide sufficient information regarding treatment and follow-up during the pandemic. Structural elements, such as access to mail and telephone support, IBD-nurse consultation, and information and disease advice from IBD specialists regarding IBD and COVID-19 are recommended to provide the most effective model of care [6, 27].

We found that the IBD treatment adherence was 98% in our study. All patients in our study received biological treatment, and many patients received infusion therapy. Despite increased concerns among IBD patients during the pandemic, other recent studies also found a similarly high treatment adherence [25, 28].

Female patients with IBD were more likely to have higher levels of anxiety than male patients. One reason for this may be the higher proportion of women on sick leave or temporarily laid off than men. The distribution of employment status and depression was similar between the genders. However, patients on sick leave or temporarily laid off were almost four times more likely to be depressed than those who were employed or were students. This may be explained by the fact that being out of work contributes to uncertainty in general and is a highly stressful situation for some.

All patients with IBD included in this study received biological treatment as monotherapy or in combination with immunosuppressants. This patient subgroup is the most vulnerable with respect to COVID-19, and changes in follow-up might create higher levels of both disease-related and general life stress. The patients receiving biological treatment comprised nearly half of the outpatients visiting our IBD unit at the hospital. Improved access to healthcare professionals with IBD specialist expertise and better facilitation of healthcare services may reduce depression and anxiety in this cohort of patients with IBD.

### Strengths and limitations

This study was conducted early during the pandemic and included a well-defined and complete cohort of outpatients with IBD on biologics from a large university hospital. The proportion of non-responders was negligible, thus ensuring the very high generalizability of our results. However, this study was conducted early in the pandemic in a single center at an University Hospital in Norway. Therefore, the results may not be generalized to a general IBD population, and the patient experiences of the pandemic situation and the identified association to anxiety and depression may be affected by this. When a tailored information from the Norwegian authorities and the health care providers is available, the patients’ experiences may be different. Therefore, a follow-up study would be recommended. At the time of the study, no validated questionnaire regarding COVID-19 restrictions had been developed, and some of our questions may have been ambiguous.

### Conclusion

COVID-19 restrictions was associated with anxiety and depression in patients with IBD undergoing biological treatment. Structural elements such as easy access to mail and telephone support, high-quality information, and disease advice from physicians regarding IBD are factors associated with lower levels of anxiety and depression. These results will help us to facilitate future healthcare services for patients with IBD and develop updated and evidence-based guidelines for routine follow-up in outpatient clinics.
